# Adaptive structure evolution and biologically plausible synaptic plasticity for recurrent spiking neural networks

**DOI:** 10.1038/s41598-023-43488-x

**Published:** 2023-10-07

**Authors:** Wenxuan Pan, Feifei Zhao, Yi Zeng, Bing Han

**Affiliations:** 1 Brain-inspired Cognitive Intelligence Lab, Institute of Automation, Chinese Academy of Sciences, Beijing, China; 2 School of Artificial Intelligence, University of Chinese Academy of Sciences, Beijing, China; 3 School of Future Technology, University of Chinese Academy of Sciences, Beijing, China; 4 Center for Excellence in Brain Science and Intelligence Technology, Chinese Academy of Sciences, Shanghai, China

**Keywords:** Machine learning, Learning algorithms, Network models

## Abstract

The architecture design and multi-scale learning principles of the human brain that evolved over hundreds of millions of years are crucial to realizing human-like intelligence. Spiking neural network based Liquid State Machine (LSM) serves as a suitable architecture to study brain-inspired intelligence because of its brain-inspired structure and the potential for integrating multiple biological principles. Existing researches on LSM focus on different certain perspectives, including high-dimensional encoding or optimization of the liquid layer, network architecture search, and application to hardware devices. There is still a lack of in-depth inspiration from the learning and structural evolution mechanism of the brain. Considering these limitations, this paper presents a novel LSM learning model that integrates adaptive structural evolution and multi-scale biological learning rules. For structural evolution, an adaptive evolvable LSM model is developed to optimize the neural architecture design of liquid layer with separation property. For brain-inspired learning of LSM, we propose a dopamine-modulated Bienenstock-Cooper-Munros (DA-BCM) method that incorporates global long-term dopamine regulation and local trace-based BCM synaptic plasticity. Comparative experimental results on different decision-making tasks show that introducing structural evolution of the liquid layer, and the DA-BCM regulation of the liquid layer and the readout layer could improve the decision-making ability of LSM and flexibly adapt to rule reversal. This work is committed to exploring how evolution can help to design more appropriate network architectures and how multi-scale neuroplasticity principles coordinated to enable the optimization and learning of LSMs for relatively complex decision-making tasks.

## Introduction

The brain is a highly heterogeneous and powerful network of tens of billions of neurons possessing unparalleled feats of cognitive functions. Traditional artificial intelligence models are predominantly built on networks with hierarchical feed-forward architectures, different from the highly recurrent connected biological network in the brain ^1^, making it difficult to match the results of natural evolution in terms of function and efficiency. Macro-scale reconstruction studies of human brain structure ^2^ confirmed the existence of a large number of non-hierarchical structures in the brain, such as modular structure ^3–6^, hub structures ^7, 8^, small-world structures ^6, 9^. These topological properties enable the brain to better coordinate multiple cognitive functions to adapt to complex and dynamic environments and are also unconventional structures missing in existing brain-inspired AI models.

**Figure 1:**
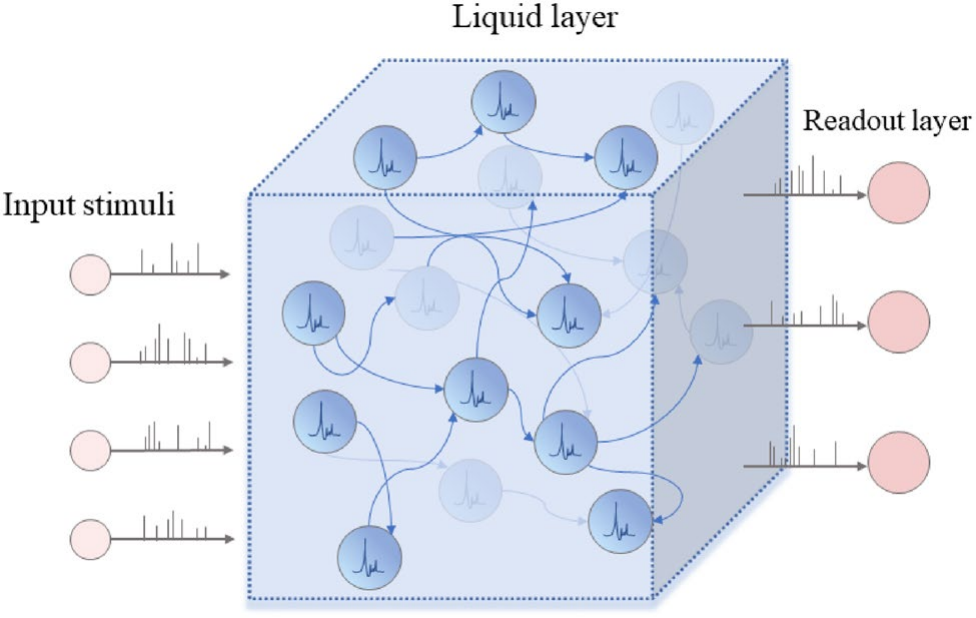
In the traditional definition of the LSM, randomly connected liquid layer neurons receive time-varying signals from external inputs and other nodes. Recursive connection patterns enable input signals to be converted into liquid layer dynamics and then abstracted by the readout layer.

Motivated by this, this work focuses on a network structure called Liquid State Machine (LSM) which can generate complex dynamics like the brain and facilitate the processing of real-time tasks. LSM ^10^ is a spiking neural network (SNN) structure that belongs to the reservoir, with randomly connected liquid layers and readout layers whose weights can be modified, as shown in Fig. 1. Reservoir computing has achieved some progress in different fields, such as speech recognition^11–13^, image recognition^14–16^, robot control^17, 18^, etc. Existing hardware designs for spiking neural networks can not only carry LSM ^19–21^, but also adaptively switch synaptic plasticity ^20^, and have been proven to be energy-efficient ^22, 23^.

Some LSM models use fixed weights for the liquid layer, probably because its complex recurrent structure is difficult to be trained and optimized, which limits the learning capability and wide application of LSM ^17, 24, 25^. Most of these existing models used gradient-based approach  ^26–30^ to train the readout layer without training the liquid layer, resulting in a gap with the real learning mechanism in the brain. Some approaches ^31–34^ tried to train the liquid layer through local synaptic plasticity such as Spike-Timing-Dependent Plasticity (STDP) ^35^ or Hebb ^36^, which is limited to simple tasks. In summary, there is still a need to explore biologically plausible learning rules applicable to LSM to optimize its liquid and readout layers.

In addition, from the structure perspective, the liquid layer is usually fixed after initialization, simply serving as a way of high-dimensional encoding. Some methods ^30, 33, 37^ inspired by deep neural networks superposed multiple LSM layers as a deep LSM to solve machine learning tasks. These approaches have not explored the studies about dynamic LSM’ structure search in order to adapt to the changing tasks. And in fact, the human brain evolved rather than followed a specific design, which is different from current common AI algorithms. Evolution allows the brain’s nervous system to be continuously optimized and eventually evolve into non-hierarchical, highly-efficient structures. Inspired by this, some studies ^25, 26, 28, 32, 38^ proposed evolutionary methods for optimizing the parameters and structures of LSM. Previous study ^17^ assessed LSM according to three LSM’s properties ^10^, and this work encoded the three LSM properties into the chromosome, and optimized the separation property (SP) as the objective function. Using SP as fitness is reasonable because it could reflect the role of evolution in the network dynamic adjustment. However, this work is limited to simple control tasks. A study ^26^ developed a three-step search method based on the genetic algorithm (GA) to search the network architecture and parameters of LSMs. Some researchers ^26, 28, 32^ directly used the experimental data set as a criterion for evaluating the fitness of LSM. These approaches lack effective exploitation of the internal dynamics of LSM.

Considering the various limitations of existing LSM’s studies mentioned above, in this paper, we present a brain-inspired LSM with evolutionary architecture and dopamine-modulated Bienenstock-Cooper-Munros (DA-BCM) Rule. We consider the optimization of LSM from structure and function respectively. *Structurally*, we optimize the architecture of liquid layer according to an evolutionary perspective to obtain a more brain-inspired effective structure with a higher separation property. *Functionally*, instead of the gradient-based method, we propose a biologically plausible learning method with the combination of local trace-based BCM^39^ synaptic plasticity and global dopamine regulation. The experimental results show that the proposed evolved DA-modulated LSM is able to learn the correct strategy faster and flexibly adapt to rules reversal on multiple reinforcement learning tasks. As reservoir computation exhibits complex dynamics consistent with activity in brain neural circuits, the evolvable LSM based on DA-BCM provides us with a powerful and biologically realistic tool to delve deeper into the learning process of complex networks. This work provides new opportunities for developing more brain-inspired complex and efficient network models, building adaptive learning frameworks, and revealing the evolutionary mechanisms of brain structures and functions.

## Methods

In this section, we first introduce the architecture and construction details of LSM. Evolutionary algorithms and DA-BCM multi-scale learning rule are sequentially used to optimize the LSM’s liquid layer connectivity patterns and all weight strengths.

### LSM architecture

#### Leaky integrate-and-fire (LIF) neuron model

Neuron model used in the proposed LSM is implemented with LIF model^40^, which can be simulated by Eq. 1:

1 $$\begin{aligned}{} & {} \tau _{\textrm{m}} \frac{d V_{\textrm{m}}(t)}{d t}=I(t)-V_{\textrm{m}}(t) \end{aligned}$$

$V_{m}$ is the voltage across the cell membrane, *I* is the external input, and $\tau _{m}=2.0$ is the membrane potential time constant. The post-synaptic neuron voltage accumulates from $V_{reset}=0$ exponentially when a current spike is received from pre-synaptic neurons, producing an output spike as soon as the threshold $V_{th}=1.0$ is reached.

#### Liquid layer initialization

In the experiment, the number of neurons in the liquid layer was set to 10*10, totaling 100. Inspired by neuron connections in the mammalian visual cortex, we set the connection probability *p* between neurons *i* and *j* to exponentially inversely proportional to the Euclidean grid distance *d*(*i*, *j*) ^41^. The closer the grid distance, the higher the connection probability, which is defined as the following:

2 $$\begin{aligned} p&=\left( e^{-\frac{1}{\lambda ^{2}}}\right) ^{d^{2}} \end{aligned}$$

$\lambda$ is the parameter that controls the average number of synaptic connections and the average distance between neurons. To prevent neurons that are too far apart from connecting, a mask matrix $M_{dis}$ is added, combined with *p* to form the weight matrix $W_l$ of the liquid layer as Eqs. 3 and 4:

3 $$\begin{aligned} W_{l}&=\alpha *M_{dis}*M_{sparse}*p \end{aligned}$$

4 $$\begin{aligned} M^{i,j}_{dis}&={\left\{ \begin{array}{ll} 1, d(i,j)<D_{th} \\ 0, d(i,j)>D_{th} \quad or\quad i=j \end{array}\right. } \end{aligned}$$

Both $M_{dis}$ and $M_{sparse}$ are binary matrices. $M_{dis}$ helps to form locally interconnected liquid layer structures. Direct self-connection is not allowed but is allowed to connect back after passing through other neurons. $M_{sparse}$ describes a sparse binary matrix in which only a randomly selected 1% of the connections have the value $M_{sparse}$ equal to 1 (sparser liquid density is to facilitate subsequent evolution operations). $D_{th}$ is a threshold that limits the range of local connections at initialization, and is the furthest neighbor a single neuron can connect to. Here is set to 6. $\alpha =4$ is a constant.

#### Readout layer initialization

To construct an effective readout layer structure, preventing many inactive neurons from being read out, we formulate the connection weight $W_{r}$ between the liquid layer and the readout layer according to the state matrix *S* as follows:

5 $$\begin{aligned}{} & {} W_{r}=\beta *M_{r}*S*w_{rand} \end{aligned}$$

$w_{rand}$ indicates a rand weight matrix. Both $M_{r}$ and *S* are binary matrices. When the readout layer has $N_r$ neurons, all the liquid layer neurons are randomly divided into $N_r$ classes, making each liquid layer neuron connected to only one readout layer neuron. The resulting mask matrix $M_{r}$ specifies which liquid layer neurons are connected to which readout layer neurons. 0 in the state matrix *S* represents that the neuron did not fire, and a 1 represents it fired. Therefore, there is no connection between the non-firing liquid neurons and all the readout neurons. $\beta =4$ is a constant. The number of liquid neurons connected to each output or input neuron is set to 4.

### Evolutionary LSM structure

**Figure 2:**
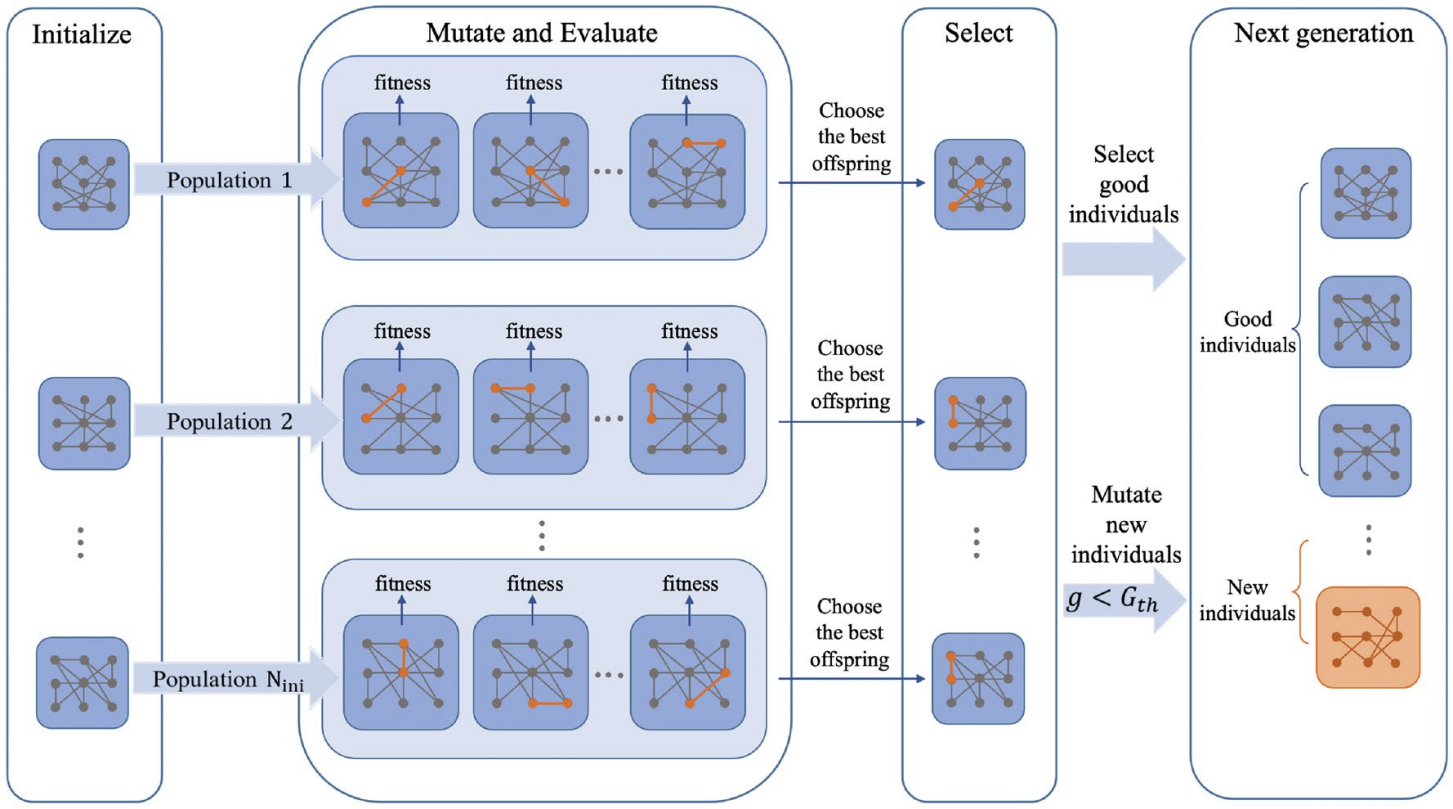
The procedure of evolutionary LSM structure.

First, we randomly initialized $N_{ini}$ LSMs to form a population *pop* with their respective liquid layer connectivity matrices as chromosomes. For each chromosome, a population of offsprings is generated, and each offspring is mutated. According to the calculated fitness function, the optimal offspring corresponding to each chromosome is selected as a new chromosome. Meanwhile, to introduce innovation, the next generation consists of all optimal offsprings and partially regenerated random individuals. Evolution continues until the $N_{opt}$ individuals with the highest fitness in $G_{th}$ generation are selected as the output of the evolutionary algorithm. Figure 2 illustrates the detailed evolutionary process.

#### Initialization

We initialize $N_{ini}$ LSM individuals, each of which is an LSM structure. A chromosome is defined as a matrix representing an individual’s liquid layer connectivity patterns:

6 $$\begin{aligned} Chrom^{i}&={\left\{ \begin{array}{ll} 1, W_{l}^{i}>0\\ 0, W_{l}^{i}=0,\quad 0\le i \le N_{ini} \end{array}\right. } \end{aligned}$$

*i* is the number of the individual, and $W_l$ is the liquid layer connection weight of the *i*th individual defined in Eq. 3.

#### Mutation

Each chromosome generates multiple offsprings (collectively a chromosome population) and mutates them: randomly select an inactive neuron (firing no spikes) among all liquid neurons and connect it with a surrounding active neuron (firing at least one spike).

#### Evaluation

The Separation Property (SP) was proposed together with the concept of LSM^10^ as a measure of performance, which calculates the separation between the internal system state trajectories produced by two different input streams: a good network has different responses to different stimuli, and the greater the input difference, the greater the difference in network firing patterns. There are many methods in current research to measure the SP of LSM, here we refer to one of them^42^ to measure the quality of the liquid layer. We first calculate a state matrix *S* (1 for fired, otherwise 0) of the liquid layer based on input and then compute the SP according to the following formula:

7 $$\begin{aligned}{} & {} SP=rank(S) \end{aligned}$$

*rank*(*S*) means the rank of matrix *S*. The larger the value, the stronger the separation property of LSM. After mutation, we calculate the separation property of offsprings obtained by mutation referring to Eq. 7 as the fitness function.

#### Selection

Based on the fitness of all offsprings $F_{offs}$, select the one with the largest fitness in the chromosome population to replace the individual. In the first $G_{th}$ generation, the next generation consists of individuals with high fitness and new individuals explored randomly as a proportion of *rate* of the entire population. After $G_{th}$ times of evolution, the new generation uses the experience of multiple iterative optimizations to select $N_{opt}$ individual with the highest fitness as the evolution output.

The algorithm process of evolving the LSM architecture is Algorithm 1.
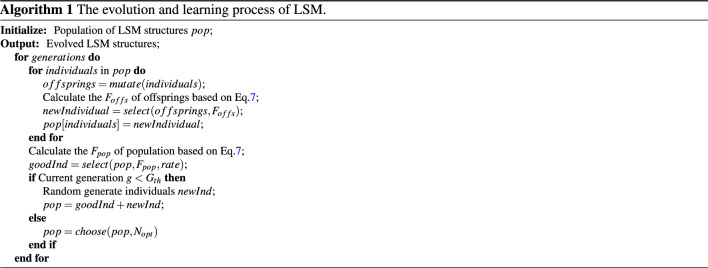


### DA-BCM for training evolved LSM

**Figure 3:**
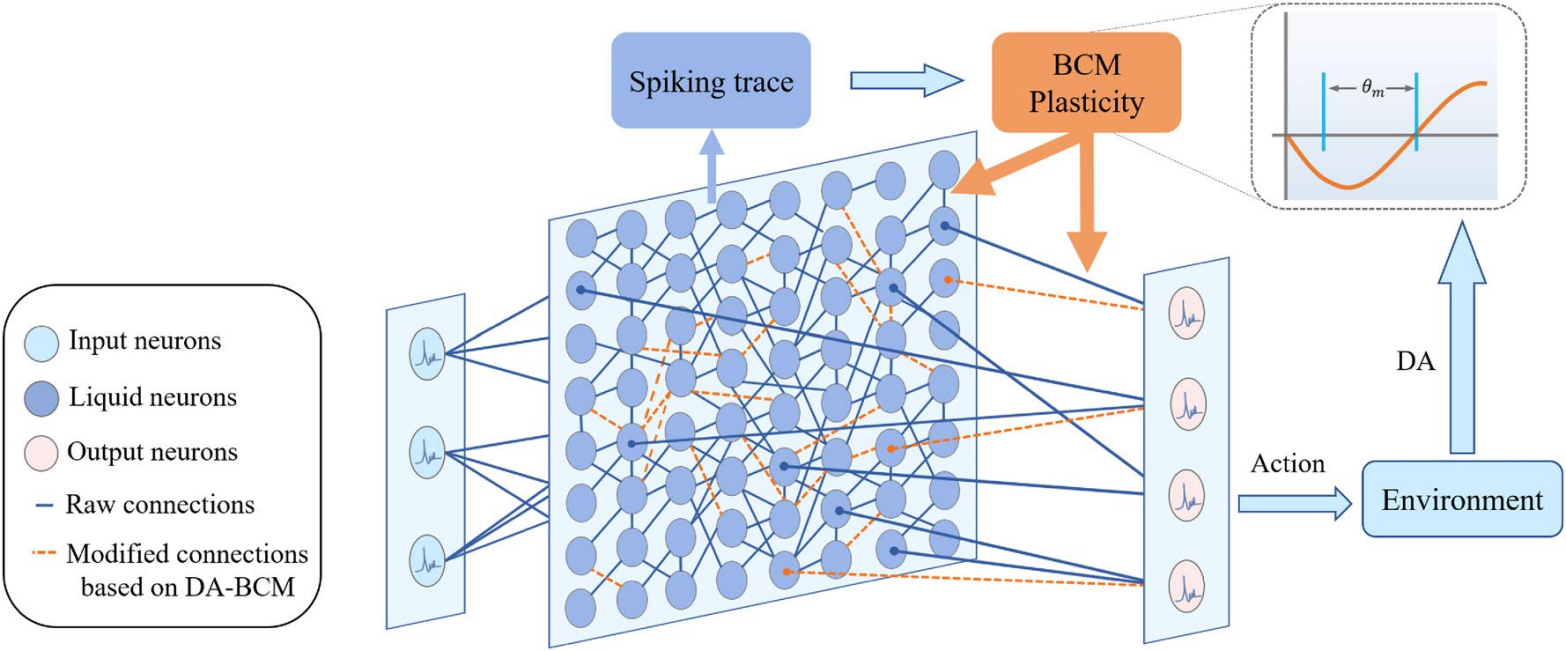
DA-BCM optimizes the synaptic weights of the evolved LSM.

After evolving LSM, we incorporated multi-scale biological-inspired learning rules such as local synaptic plasticity and global dopamine regulation for optimizing synaptic strength. As shown in Fig. 3, the learning process updates the connection weights within the liquid layer and between the readout layers according to local BCM plasticity ^39^ and global dopamine regulation. Finally, the readout neuron with the most firing rates is selected as the action.

#### Local BCM synaptic plasticity

Since gradient-based learning rules are inconsistent with biological reality, we employed a more biologically plausible mechanism of synaptic plasticity: BCM rules^39^, combined with dopamine (DA) global regulation to simulate the effects of reward and historical memory on behavior, encoding readout neurons target spatio-temporal dynamics. BCM was first used to explain how cortical neurons simultaneously undergo LTP or LTD depending on the different regulatory stimulation protocols applied to pre-synaptic neurons^43^. According to BCM, the activity of the postsynaptic neuron strengthens the connection, and the activity experience of the postsynaptic neuron determines the dynamic correction of the threshold. The synaptic strength update rule for the activity of pre- and post-synaptic neurons is as follows:

8 $$\begin{aligned} \frac{d m(t)}{d t}=\phi (e_{post}^{t}) e_{pre}^{t}-\epsilon m(t) \end{aligned}$$

*m* is the weight between pre- and post-synaptic neurons. $\epsilon$ is a coefficient that decays uniformly over time. $\phi$ is the BCM modification function that adjusts according to the neural spiking trace of the postsynaptic neuron, incorporating a sliding activity-dependent modification threshold $\theta _{m}$ to allow bidirectional synaptic modification. $e_{pre}^{t}$ is the spiking trace of the presynaptic neuron at time *t* and $e_{post}^{t}$ is the spiking trace of the postsynaptic neuron at time *t*, which are calculated as:

9 $$\begin{aligned} e_{pre}^{t}=\tau _{bcm} e_{pre}^{t-1}+o_{pre}^{t} \end{aligned}$$

10 $$\begin{aligned} e_{post}^{t}=\tau _{bcm} e_{post}^{t-1}+o_{post}^{t} \end{aligned}$$

Where $o_{pre}^{t}$ and $o_{post}^{t}$ denote the spikes of pre- and post-synaptic neurons, respectively. $\tau _{bcm}$ is the time decay constant which is set to 3.0. $\phi$ is defined as:

11 $$\begin{aligned} \phi (e)=e (e-\theta _{m}) \end{aligned}$$

The sliding threshold $\theta _{m}$ is dynamically updated according to the average value of the trace *e* over a period of time 10.

#### Global dopamine regulation

Previous study^44^ proposed the “reward prediction error hypothesis” that dopamine neurons encode reward and punishment signals during interacting with the environment. Related studies have introduced the learning rules of reward regulation into deep spiking neural networks ^45^ and multi-brain regions coordinated SNNs ^46, 47^. Further, reward-modulated STDP ^48–50^ integrates dopamine regulation and STDP could solve the problem of credit assignment in order to obtain more reward.

Here, inspired by the neural mechanisms of dopamine regulation, we propose a DA-BCM learning rule that integrates long-term dopamine regulation and local BCM synaptic plasticity. When receiving an external reward signal, dopamine forms a global long-term regulation, combining with BCM plasticity to adaptively adjust synaptic strength for the liquid layer and readout layer. The DA-BCM learning rule is as follows:

12 $$\begin{aligned} \frac{d m(t)}{d t}&=DA*(\phi (e_{post}^{t}) e_{pre}^{t}-\epsilon m(t)) \end{aligned}$$

Here, *DA* stands for dopamine signal. As shown in Fig. 3, the reward information *DA* in the environment is fed back to the local synaptic plasticity mechanism (Eq. 12) in the form of dopamine, forming a global regulatory effect.

## Results

### The evolution and learning process

In this paper, the liquid layer connectivity of LSM is first evolved, and then the liquid layer and readout layer are optimized based on DA-BCM to realize the online decision-making of the whole model (including input layer, liquid layer and readout layer). Evolution randomly initializes $N_{ini}=100$) individuals according to the inputs of different tasks, and then randomly mutates multiple offspring during the mutation process, finally selecting the optimal $N_{opt}=20$ structures for decision making. All experimental results in this work are based on the average of the network structures obtained from multiple random evolution to ensure accuracy and fairness.

Experiments show that evolved individuals are superior to randomly generated models in efficiency. Based on the evolved structures, the $N_{opt}$ individuals (agents) are then placed in a specific environment, where the next step action is determined according to the LSM’s output. DA-BCM rule dynamically adjusts the strength of LSM’s weights through the reward signal fed back by the environment, enabling the agent to learn and survive better in the environment. Model performance is evaluated according to the average cumulative reward of $N_{opt}$ individuals within $T=500$ steps, which is calculated as follows:

13 $$\begin{aligned} R&= \frac{{\textstyle \sum _{i}^{N_{opt}}} {\textstyle \sum _{t}^{T}}DA_{t}^{i}}{N_{opt}} \end{aligned}$$

$DA_{t}^{i}$ represents the reward obtained by individual *i* at step *t*. Therefore, *R* represents the average reward of all individuals.

**Figure 4:**
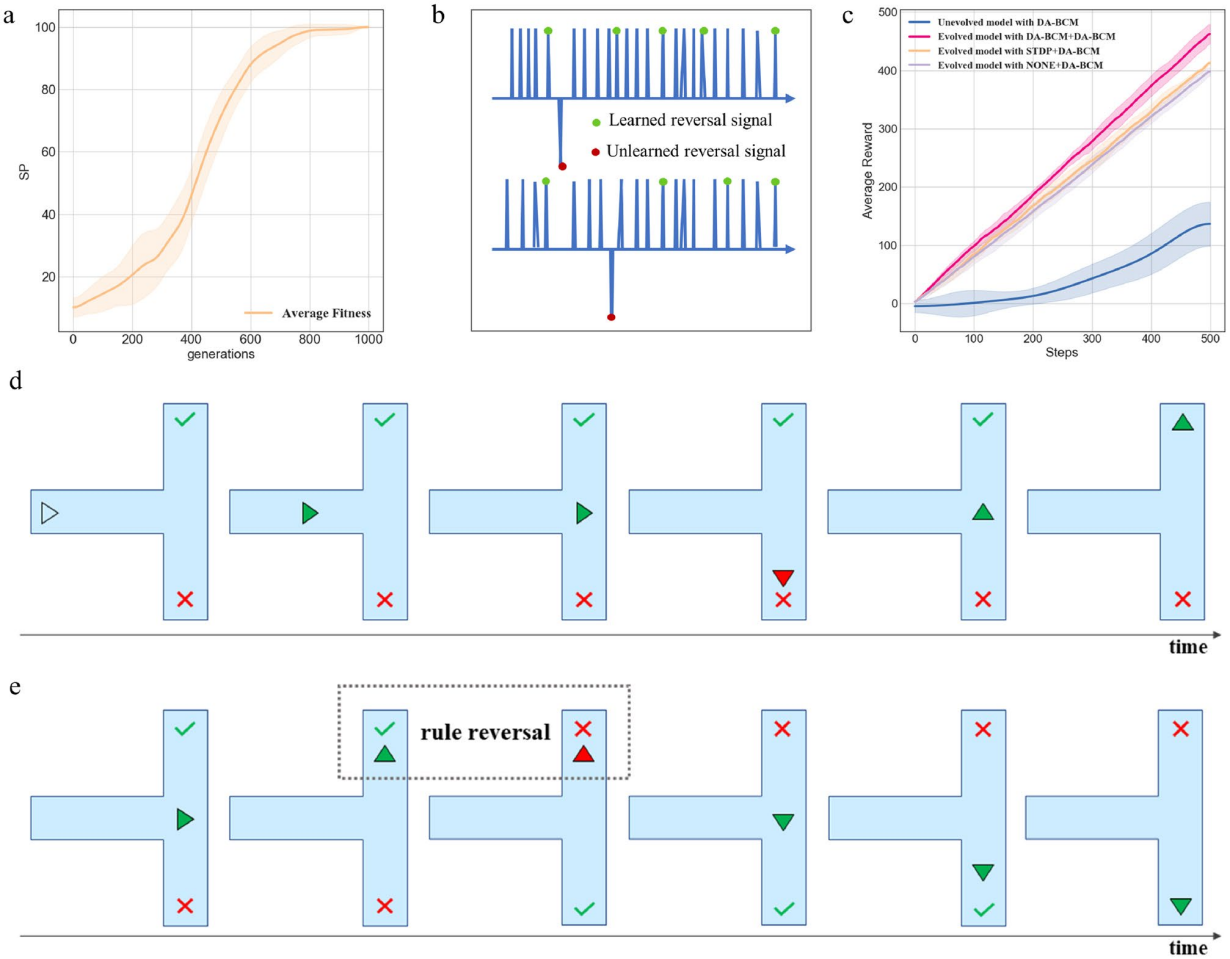
Experimental results on T-maze. (**a**) The separation property of evolving LSMs which is calculated from the average of all individuals in population. (**b**) Reversal learning results. Green dots indicate that the agent has obtained food, and red dots indicate poison. (**c**) Performance of LSMs (applying different learning rules). Evolved model results are the average performance of $N_{opt}$ individuals, unevolved model results are the average performance of multiple runs. (**d**) The agent learns to change behavior guided by DA regulation. (**e**) When the rule is reversed, the agent learns to avoid the poison after being punished once.

### Validation on T-maze

#### Experiment configuration

We constructed a T-shaped maze (two ends of the maze are food and poison, respectively). The maze can be regarded as the size of 3x5 (the size of three grids in the vertical direction and the size of five grids in the horizontal direction), as shown in Fig. 4d,e. Three input neurons representing the agent’s observations in three directions (maybe walls, roads, food, poison) feed information into the evolved LSM, and the agent performs actions (forward, left, right) based on the output. For example, if walls, roads, food, and poison are represented by 0, 1, 2, and 3 respectively, then (0, 1, 0) means that the agent sees the wall on the left, the wall on the right, and the road ahead. The output (1, 0, 0) means the action taken is left, (0, 1, 0) means forward, and (0, 0, 1) means right.

The distance difference between the agent and the food before and after executing the behavior is defined as $dis_{m}$, then the reward function for the T-maze task is:

14 $$\begin{aligned}{} & {} DA= {\left\{ \begin{array}{ll} 3,&{}get\ food\\ -3,&{}get\ poison\\ 1,&{}dis_{m}<0\\ -1,&{}dis_{m}\ge 0\\ \end{array}\right. } \end{aligned}$$

An energy mechanism is set up to prevent the agent from wandering or standing still (i.e.hitting the wall all the time) in the maze. Each round is counted from the starting point to the endpoint, where learning time is limited to a certain number of steps, after which the exploration process will be restarted. When the agent receives positive rewards consecutively (more than ten times), the positive and negative rewards in the maze will exchange positions with a certain probability (set to 0.3 here), thus verifying the ability of the model to adapt to reverse learning.

#### Results on T-maze

The fitness change of the evolved $N_{opt}$ individuals is shown in Fig. 4a, which can gradually evolve to reach the maximum value, verifying the evolutionary algorithm’s effectiveness. Comparing evolved and unevolved models in Fig. 4c, we could find that structure evolution improves the learning efficiency and performance of LSM models. Models’ performance is calculated using the average reward value of $N_{opt}$ individuals over a period of time $T=500$. Figure 4d shows how evolved LSMs with DA-BCM learning rule help the agent to find where the food is. Along the way, reward signals of environmental feedback (shown in green and red, respectively) guide agent behavior through dopamine modulation.

*Reversal Learning* During the learning process, the agent showed the ability to flexibly adapt to the reversion of the rules. As shown in Fig. 4b, after taking the poison for the first time and being punished, the agent can avoid the poison no matter how the positions of the poison and food are changed, which means that agent has the ability to reversal learning and can flexibly handle changes in the environment. Simulation results shown in Fig. 4e indicate that the agent exhibits the ability of reversal learning.

*Ablation analysis* Ablation experiments further evaluate the effect of DA-BCM learning rules by applying STDP and DA-BCM to the liquid and readout layers to explore the effect of different learning rules on LSM performance. As shown in Table 1 and Fig. 4c, the evolved LSM with liquid and readout layers trained by DA-BCM achieves the best performance and significantly outperforms other models. The worst among all methods is the evolved LSM trained with unsupervised STDP, indicating that the model without any environmental feedback to guide LSM dynamics cannot gain knowledge, causing the average reward *R* to fluctuate over time with no cumulative trend. Besides, the none+DA-BCM and STDP+DA-BCM (the front of “+” represents the liquid layer learning rule, and the back represents the readout layer learning rule) models achieve similar good performance, indicating that the regulation of DA-BCM at the readout layer can help the model to learn the rules of the environment. The STDP+DA-BCM and DA-BCM+DA-BCM are superior to the none+DA-BCM, which indicates that optimizing the weights of liquid layer is more effective than fixing their weights. Further, the outstanding advantage of DA-BCM+DA-BCM illustrates that our proposed biologically plausible DA-BCM training method can outperform untrained or STDP-trained models, helping to evolve LSM’s structure and learn the environment information more efficiently.

**Table 1:** Results of ablation experiments on T-maze.

Structure	Liquid Layer	Readout Layer	Performance
Evolved	STDP	STDP	− 0.9±5.54
Unevolved	DA-BCM	DA-BCM	162.0±12.44
Evolved	none	DA-BCM	399.44±5.99
Evolved	STDP	DA-BCM	414.75±1.41
Evolved	DA-BCM	DA-BCM	464.4±5.15

**Table 2:** Reward function for Flappy Bird.

Current state	last state $=$ current state		last state $\ne$ current state
	$dis_f<0$	$dis_f \ge 0$	
0 or 1	6	6	6
2 or 3	3	$-$5	$-$3
4 or 5	3	$-$8	$-$5
6 or 7	3	$-$3	$-$3
8	$-$100	$-$100	$-$100

### Validation on Flappy Bird

#### Experiment configuration

**Figure 5:**
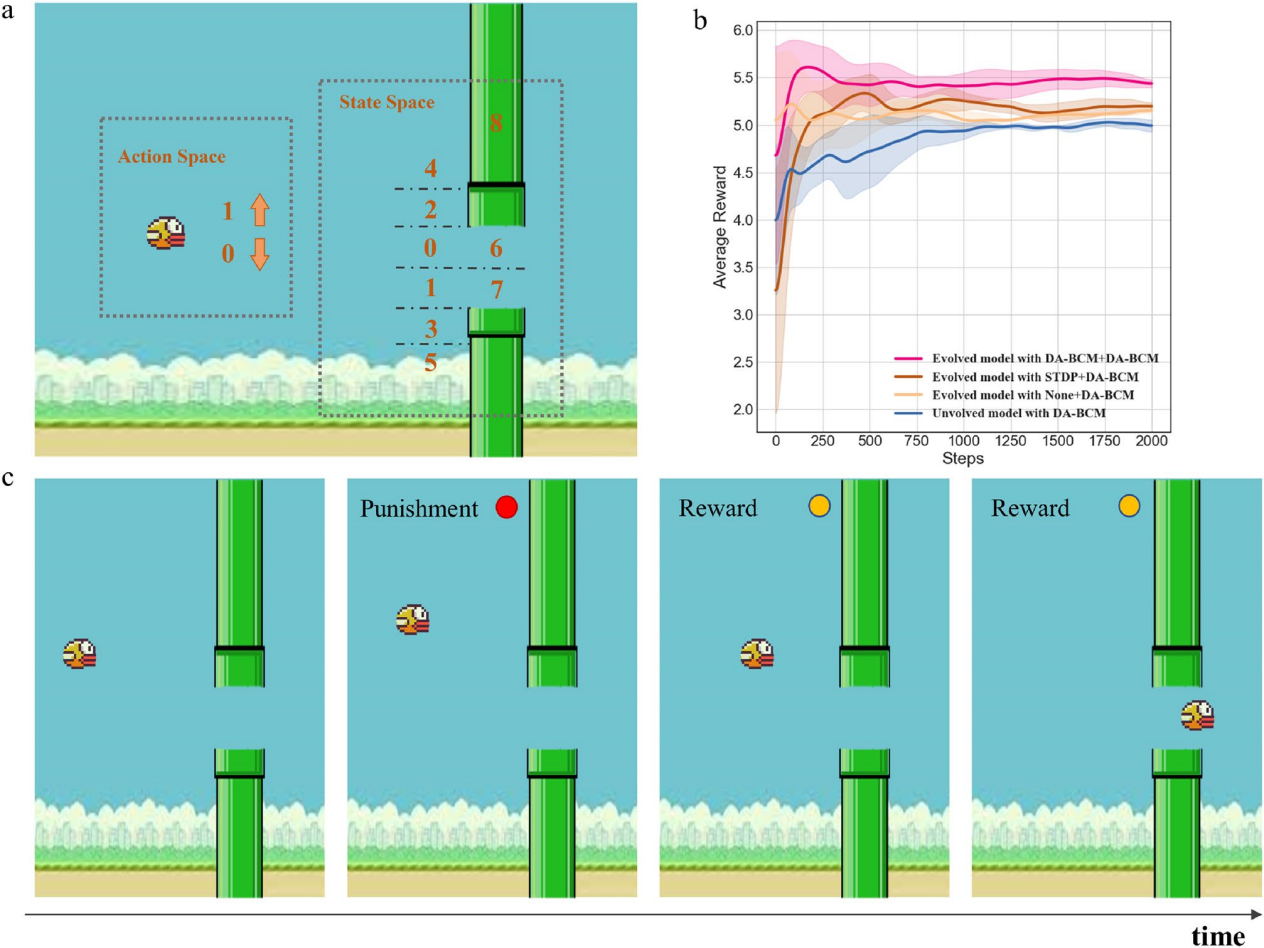
Experimental results on Flappy Bird. (**a**) The setup of state space and action space. The whole space is divided into 9 states, where 6 and 7 are the ultimate goals to be achieved. (**b**) The final performance of all models in the Flappy Bird environment. Evolved model results are the average performance of $N_{opt}$ individuals, unevolved model results are the average performance of multiple runs. (**c**) Agents avoid mistakes under the guidance of reward signals.

Flappy Bird is a game in which the player controls the bird to move between the gaps of many pipes without colliding as much as possible. The settings of state space and action space are shown in the Fig. 5a, the size of action space is 2 (i.e. up or down). We divide the state space into 9 parts according to the positional relationship between the bird and the pipe. The state of the bird and its action at the next moment are the input and output of the evolved LSM. For example, if the agent is in state 2, then the input is (0, 0, 1, 0, 0, 0, 0, 0, 0, 0). Output (1, 0) represents action 0, (0, 1) represents action 1. The positive reward is used to encourage the bird to pass the pipes (i.e. the gap between the upper and lower pipes), and the negative reward is used to punish the bird for staying away from the exit of the pipes. The reward is learned by LSM for the adjustment of synaptic strength (based on DA-BCM). When the bird collides with the pipe, the game is over.

Table 2 illustrates the definition of reward function (*DA*) for Flappy Bird. The reward is determined according to current state and last state, and the distance difference $dis_f$ between the bird and the center of pipe before and after executing a selected behavior. The maximum positive reward is given when the bird is in state 0 or 1. A slightly smaller positive reward is used to encourage shorter distances ($dis_f<0$) to the target location (i.e. empty space between pipes). Correspondingly, if the distance becomes longer ($dis_f \ge 0$), a negative reward is given. The largest negative reward is used to punish hitting the pipe. Models’ performance is calculated using the average reward value of $N_{opt}$ individuals over a period of time steps $T=2000$.

#### Results on Flappy Bird

To verify the validity of the proposed model, we compared the unevolved LSM with liquid layer and the readout layer both trained by DA-BCM (unevolved model with DA-BCM in Fig. 5b), the evolved LSM with non-trained liquid layer and DA-BCM trained readout layer (evolved model with None+DA-BCM in Fig. 5b), the evolved LSM with STDP-trained liquid layer and DA-BCM trained readout layer (evolved model with STDP+DA-BCM in Fig. 5b), and the evolved LSM with DA-BCM trained liquid layer and DA-BCM trained readout layer (evolved model with DA-BCM+DA-BCM in Fig. 5b), respectively. Figure 5b depicts the average reward curves (Gaussian smoothed) for different models. It is obvious that evolved model with DA-BCM+DA-BCM achieves the best results, while unevolved method is inferior to the evolved methods. Comparing the optimization methods for liquid layer, STDP slightly outperforms the untrained method, while DA-BCM can further bring improvements. Figure 5c shows that our proposed model can guide the bird to fly smoothly through the pipe via dopamine modulation.

**Table 3:** Results of ablation experiments on Flappy Bird.

Structure	Liquid layer	Readout layer	Performance
Evolved	STDP	STDP	− 54.77±59.7
Unevolved	DA-BCM	DA-BCM	4.97±0.04
Evolved	none	DA-BCM	5.29±0.00
Evolved	STDP	DA-BCM	5.36±0.02
Evolved	DA-BCM	DA-BCM	5.43±0.03

The detailed final performances (average reward *R* and its variance) of different methods are listed in Table 3. LSM trained only with STDP could not finish this task, failing to get positive feedback from the environment, causing the bird to hit the pipe from the start and the game to stop. Each component of the proposed model such as evolution, DA-BCM in the liquid layer and readout layer enables the LSM network to learn faster and better. Thus, we can conclude that our work brings outstanding superiority to optimizing the LSM from the structural and functional perspectives.

**Figure 6:**
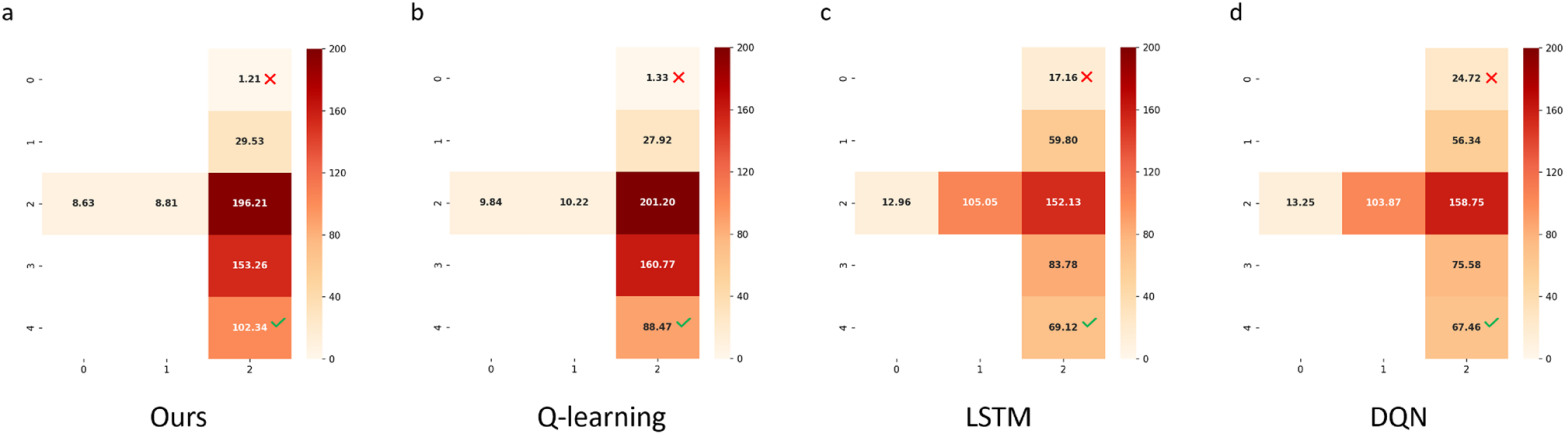
Policy maps in T-maze of four models. The value in the figure represents the number of times the agent reaches a certain state within $T=500$ steps (average of multiple experiments).

**Figure 7:**
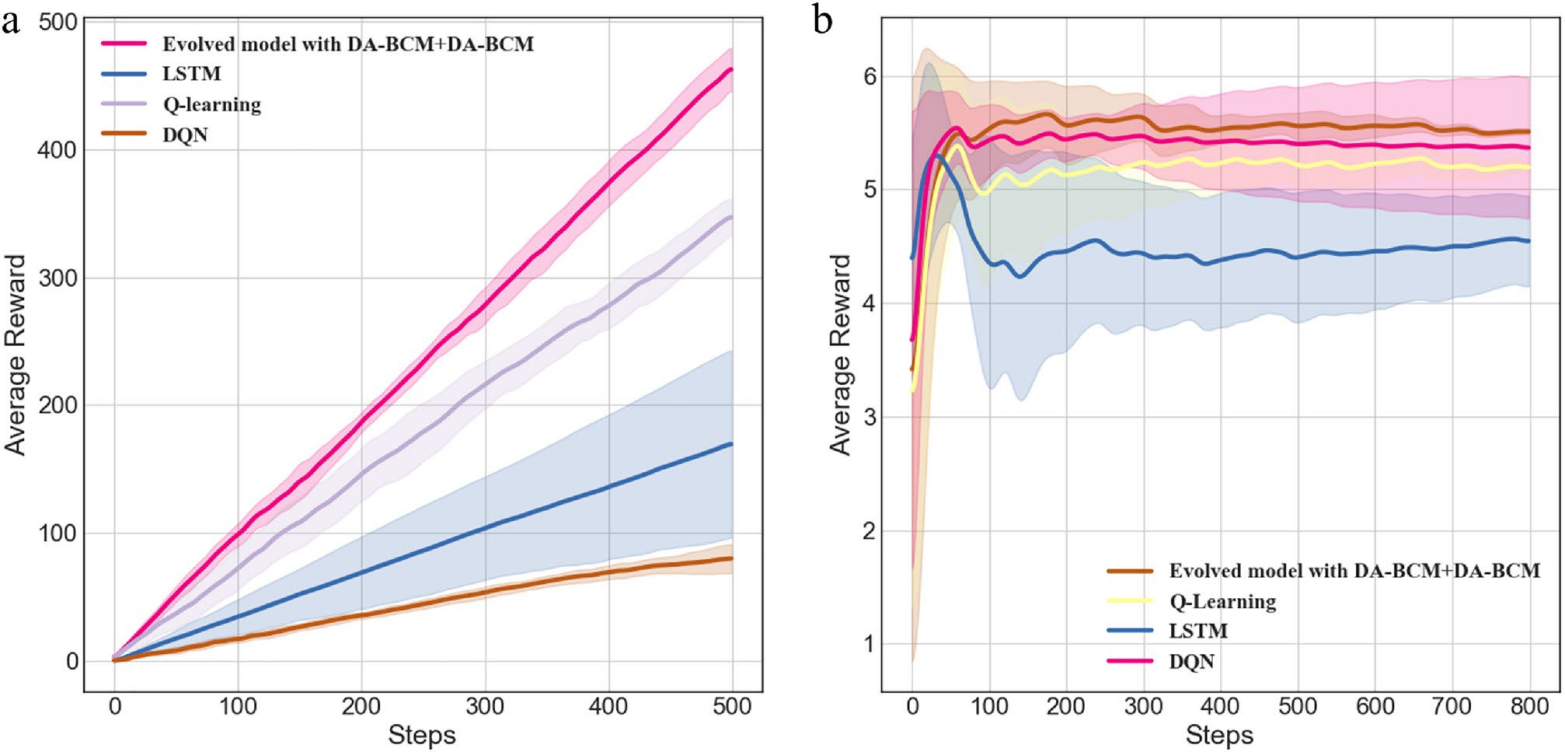
Comparative experimental results on two tasks. (**a**) Comparison results of four models in T-maze. (**b**) Comparison results of four models in Flappy Bird.

## Discussion

Evolution has not only designed the brain’s general connectivity patterns but has also optimized a multi-scale plasticity coordinated learning rule, endowing the brain with the ability to flexibly adapt to reversal learning and enabling efficient online learning. Inspired by this, this paper proposed a structurally and functionally optimized LSM model that incorporates adaptive structural evolution and biologically plausible DA-BCM learning rule. Experimental results demonstrated that the structural evolution of the liquid layer and the DA-BCM regulation of the liquid layer and the readout layer significantly improved multiple decision-making tasks.

Most existing works ^26–34^ used backpropagation-based methods (which are suitable for hierarchical networks) to optimize the readout layer without considering the optimization of the liquid layer, or only adopted unsupervised STDP to optimize the liquid layer. Our model proposed a DA-BCM learning rule for both the liquid layer and the readout layer, which shows more biologically plausible. In addition, unlike existing structural search methods that directly search for the highest-performing structure, we took inspiration from the evolutionary mechanism and optimized the structure of the LSM according to its internal properties. Here, we would like to compare our approach with other reinforcement learning models, including the classical Q-learning ^51^ ,DQN ^52^, and LSTM ^53^ (learning via policy gradient algorithm) with recurrent structure.

In LSTM configuration, the network consists of one layer of LSTM with 128 hidden neurons and one fully connected layer. The Bellman equation Q-learning uses as Eq. 15, where $\gamma =0.9$, $\alpha =0.1$. Agent’s action is selected according to the $\epsilon$-greedy algorithm ($\epsilon =0.8$), which means that there is a probability of 0.2 for each selection to explore the action space randomly. The reward discount value $\gamma$ and learning rate $\alpha$ are set to 0.99 and 0.1, respectively, in DQN. The loss function of the Q network is constructed in the form of mean square error, as shown in Eq. 16. The DQN network, which is fully connected, consists of three layers, the input layer, the hidden layer (with a size of 50), and the output layer. In Eq. 16$\gamma$ is set to 0.86. Learning rate in the used adam optimizer is set to 0.1.

For fairness, multiple experiments are performed for each comparison algorithm, and the performance is averaged. The results for LSTM, Q-learning, and DQN are averaged over multiple runs ($n=20$), where LSTM and DQN run 1000 episodes each.

15 $$\begin{aligned} Q(s, a)&=Q(s, a)+\alpha \left[ R(s, a)+\gamma \max _{a^{\prime }} Q^{\prime }\left( s^{\prime }, a^{\prime }\right) -Q(s, a)\right] \end{aligned}$$

16 $$\begin{aligned} \omega ^{*}&=\arg \min _\omega \frac{1}{2 N} \sum _{i=1}^N\left[ Q_\omega \left( s_i, a_i\right) -\left( r_i+\gamma \max _{a^{\prime }} Q_\omega \left( s_i^{\prime }, a^{\prime }\right) \right) \right] ^2 \end{aligned}$$

Figure 6 shows policy maps of the agent under the four models in the T-maze experiment. It can be seen that the well-performing model (Fig. 6a, our model) learns to avoid poison and obtain food faster. And poorly performing models not only get more poison, but hang around along the way.

Table 4 and Fig. 7 compare the average reward of the evolved $N_{opt}$ individuals under different learning rule applications in detail. From the results, it can be seen that the efficiency of our proposed model is better than the comparison algorithms in terms of both mean and stability (variance). In fact, by combining Table 3 and Table 4, it can be found that three evolutionary LSMs (DA-BCM+DA-BCM, STDP+DA-BCM, none+ DA-BCM) outperform LSTM and Q-learning in two tasks. We can also see that on the T-maze task, the performance of LSTM and DQN are significantly weaker than other models, and the variance of LSTM is very large, which may be caused by too many parameters that bring overfitting in a small sample learning task. In Flappy Bird, although DQN performance is better than LSTM and Q-Learning, the variance is very large. The overall efficiency is not as good as our model.

**Table 4:** The final performance (average reward R and its variance) for the T-maze and Flappy Bird tasks and computational cost of different methods.

Learning methods	T-maze	Flappy Bird	Computational cost
Q-learning ^51^	348.4±12.81	5.19±0.04	84
LSTM ^53^	169.95±72.61	4.54±0.40	68483
DQN ^52^	80.1±11.68	5.36±0.62	403
Ours	464.4±5.15	5.43±0.03	81.92

*Computational cost analysis* We also consider the impact of the computational cost of the model on fairness. Take T-maze for example. In our experiments, the three-layer LSTM needs to take into account the weights of 4 gates, so the total number of trainable weights is 68483. For Q-learning, only a state-action table of size $28*3=84$ needs to be stored. The number of trainable weights of the fully connected network inside the three-layer DQN is $4*50+50+50*3+3=403$. As for the LSM model we proposed, considering that the connection density of the evolved model is less than 2%, the number of connections of the liquid layer is up to about $64*64*0.02=81.92$. Including the number of connections between the liquid layer and the input and output, the total number of parameters is about 109.92 on average, of the same magnitude as Q-learning. Therefore, the computational cost of our proposed model belongs to a low level compared to DQN and LSTM.

To sum up, this work breaks through the fixed deep hierarchical network structure that relies on BP optimization used in AI, and develops a multi-scale biological plasticity coordinated learning rule (instead of BP) and an efficient structure evolution for LSM. Because the proposed model borrows the information processing mechanism of the brain from the structure and function, it is more biologically plausible, more flexible and efficient, and naturally more suitable for developing human-like cognitive intelligence. Although this paper demonstrates the superiority of our proposed evolutionary LSM model in terms of model efficiency and computational cost, there are still some limitations. For instance, while reservoirs trained with gradient methods ^26–34^ are typically limited to the readout layer, applying them to the liquid layer remains challenging. Further exploration of gradient-based approaches suitable for the reservoir architecture may be a direction to improve efficiency. There is still more room for exploration for developing brain-inspired models in learning algorithms, and many neural mechanisms are waiting for us to investigate and fully apply in the AI field. This paper focuses on the small sample learning environment. Other application scenarios can also be used to further explore more energy-efficient self-organizing brain-inspired evolutionary spiking neural networks.

## Data Availability

All original code has been deposited at https://github.com/BrainCog-X/Brain-Cog/tree/main/examples/Structure_Evolution/Adaptive_lsm.

## References

[1] Sussillo D, Abbott L (2009). Generating coherent patterns of activity from chaotic neural networks. Neuron.

[2] Suárez LE, Richards BA, Lajoie G, Misic B (2021). Learning function from structure in neuromorphic networks. Nat. Mach. Intell..

[3] Chen ZJ, He Y, Rosa-Neto P, Germann J, Evans AC (2008). Revealing modular architecture of human brain structural networks by using cortical thickness from MRI. Cereb. Cortex.

[4] Betzel RF (2017). The modular organization of human anatomical brain networks: Accounting for the cost of wiring. Netw. Neurosci..

[5] Liu Z-Q, Zheng Y-Q, Misic B (2020). Network topology of the marmoset connectome. Netw. Neurosci..

[6] Vertes PE (2012). Simple models of human brain functional networks. Proc. Natl. Acad. Sci..

[7] Towlson EK, Vertes PE, Ahnert SE, Schafer WR, Bullmore ET (2013). The rich club of the C. elegans neuronal connectome. J. Neurosci..

[8] Van Den Heuvel MP, Kahn RS, Goñi J, Sporns O (2012). High-cost, high-capacity backbone for global brain communication. Proc. Natl. Acad. Sci..

[9] Liao X, Vasilakos AV, He Y (2017). Small-world human brain networks: Perspectives and challenges. Neurosci. Biobehav. Rev..

[10] Maass W, Natschläger T, Markram H (2002). Real-time computing without stable states: A new framework for neural computation based on perturbations. Neural Comput..

[11] Goodman, E. & Ventura, D. Spatiotemporal pattern recognition via liquid state machines. In *The 2006 IEEE International Joint Conference on Neural Network Proceedings*, 3848–3853 (IEEE, 2006).

[12] Zhang Y, Li P, Jin Y, Choe Y (2015). A digital liquid state machine with biologically inspired learning and its application to speech recognition. IEEE Trans. Neural Netw. Learn. Syst..

[13] Wu J, Chua Y, Zhang M, Li H, Tan KC (2018). A spiking neural network framework for robust sound classification. Front. Neurosci..

[14] Panda P, Srinivasa N (2018). Learning to recognize actions from limited training examples using a recurrent spiking neural model. Front. Neurosci..

[15] Zhang W, Li P (2019). Information-theoretic intrinsic plasticity for online unsupervised learning in spiking neural networks. Front. Neurosci..

[16] Srinivasan G, Panda P, Roy K (2018). Spilinc: Spiking liquid-ensemble computing for unsupervised speech and image recognition. Front. Neurosci..

[17] Hourdakis E, Trahanias P (2013). Use of the separation property to derive liquid state machines with enhanced classification performance. Neurocomputing.

[18] Urbain G, Degrave J, Carette B, Dambre J, Wyffels F (2017). Morphological properties of mass-spring networks for optimal locomotion learning. Front. Neurorobot..

[19] Roy K, Jaiswal A, Panda P (2019). Towards spike-based machine intelligence with neuromorphic computing. Nature.

[20] Bianchi S (2023). A self-adaptive hardware with resistive switching synapses for experience-based neurocomputing. Nat. Commun..

[21] Ku, B. W. et al. Design and architectural co-optimization of monolithic 3d liquid state machine-based neuromorphic processor. In *Proceedings of the 55th Annual Design Automation Conference*, 1–6 (2018).

[22] Wang, S., Kang, Z., Wang, L., Li, S. & Qu, L. A hardware aware liquid state machine generation framework. In *2021 IEEE International Symposium on Circuits and Systems* (ISCAS), 1–5 (IEEE, 2021).

[23] Wang L (2022). LSMCore: A 69k-synapse/mm 2 single-core digital neuromorphic processor for liquid state machine. IEEE Trans. Circuits Syst. I Regul. Pap..

[24] Ivanov V, Michmizos K (2021). Increasing liquid state machine performance with edge-of-chaos dynamics organized by astrocyte-modulated plasticity. Adv. Neural Inf. Process. Syst..

[25] Ju H, Xu J-X, Chong E, VanDongen AM (2013). Effects of synaptic connectivity on liquid state machine performance. Neural Netw..

[26] Tian S (2021). A neural architecture search based framework for liquid state machine design. Neurocomputing.

[27] Wijesinghe P, Srinivasan G, Panda P, Roy K (2019). Analysis of liquid ensembles for enhancing the performance and accuracy of liquid state machines. Front. Neurosci..

[28] Iranmehr E, Shouraki SB, Faraji MM, Bagheri N, Linares-Barranco B (2019). Bio-inspired evolutionary model of spiking neural networks in ionic liquid space. Front. Neurosci..

[29] Han, Y., Yu, T., Cheng, S. & Xu, J. cascade spiking neuron network for event-based image classification in noisy environment. preprint (2021). 10.36227/techrxiv.16571043.v1.

[30] Soures N, Kudithipudi D (2019). Deep liquid state machines with neural plasticity for video activity recognition. Front. Neurosci..

[31] Lin X (2021). A brain-inspired computational model for spatio-temporal information processing. Neural Netw..

[32] Xue F, Hou Z, Li X (2013). Computational capability of liquid state machines with spike-timing-dependent plasticity. Neurocomputing.

[33] Wang, Q. & Li, P. D-LSM: Deep liquid state machine with unsupervised recurrent reservoir tuning. In *2016 23rd International Conference on Pattern Recognition (ICPR)*, 2652–2657, 10.1109/ICPR.2016.7900035(IEEE, Cancun, 2016).

[34] Maes A, Barahona M, Clopath C (2020). Learning spatiotemporal signals using a recurrent spiking network that discretizes time. PLoS Comput. Biol..

[35] Bi G-Q, Poo M-M (1998). Synaptic modifications in cultured hippocampal neurons: Dependence on spike timing, synaptic strength, and postsynaptic cell type. J. Neurosci..

[36] Amit DJ, Brunel N, Tsodyks M (1994). Correlations of cortical Hebbian reverberations: Theory versus experiment. J. Neurosci..

[37] Das, D., Bhattacharya, S., Pal, U. & Chanda, S. PLSM: A parallelized liquid state machine for unintentional action detection. arXiv:2105.09909 [cs] (2021). ArXiv: 2105.09909.

[38] Reynolds, J. J. M., Plank, J. S. & Schuman, C. D. Intelligent reservoir generation for liquid state machines using evolutionary optimization. In *2019 International Joint Conference on Neural Networks (IJCNN)*, 1–8, 10.1109/IJCNN.2019.8852472(IEEE, Budapest, Hungary, 2019).

[39] Bienenstock EL, Cooper LN, Munro PW (1982). Theory for the development of neuron selectivity: Orientation specificity and binocular interaction in visual cortex. J. Neurosci..

[40] Abbott LF (1999). Lapicque’s introduction of the integrate-and-fire model neuron (1907). Brain Res. Bull..

[41] Kaiser M, Hilgetag CC, Van Ooyen A (2009). A simple rule for axon outgrowth and synaptic competition generates realistic connection lengths and filling fractions. Cereb. Cortex.

[42] Legenstein R, Maass W (2007). Edge of chaos and prediction of computational performance for neural circuit models. Neural Netw..

[43] Coesmans M, Weber JT, De Zeeuw CI, Hansel C (2004). Bidirectional parallel fiber plasticity in the cerebellum under climbing fiber control. Neuron.

[44] Schultz W, Dayan P, Montague PR (1997). A neural substrate of prediction and reward. Science.

[45] Mozafari M, Ganjtabesh M, Nowzari-Dalini A, Thorpe SJ, Masquelier T (2019). Bio-inspired digit recognition using reward-modulated spike-timing-dependent plasticity in deep convolutional networks. Pattern Recogn..

[46] Zhao F, Zeng Y, Guo A, Su H, Xu B (2020). A neural algorithm for drosophila linear and nonlinear decision-making. Sci. Rep..

[47] Zhao F, Zeng Y, Xu B (2018). A brain-inspired decision-making spiking neural network and its application in unmanned aerial vehicle. Front. Neurorobot..

[48] Izhikevich EM (2007). Solving the distal reward problem through linkage of STDP and dopamine signaling. Cereb. Cortex.

[49] Fang H, Zeng Y, Zhao F (2021). Brain inspired sequences production by spiking neural networks with reward-modulated STDP. Front. Comput. Neurosci..

[50] Frémaux N, Gerstner W (2016). Neuromodulated spike-timing-dependent plasticity, and theory of three-factor learning rules. Front. Neural Circuits.

[51] Watkins CJ, Dayan P (1992). Q-learning. Mach. Learn..

[52] Mnih, V. et al. Playing atari with deep reinforcement learning. arXiv preprint arXiv:1312.5602 (2013).

[53] Hochreiter S, Schmidhuber J (1997). Long short-term memory. Neural Comput..

